# Feasibility and Acceptability of Assessing Personal Care Product Use and Exposure to Endocrine-Disrupting Chemicals Among Black and Hispanic Breast Cancer Survivors: A Pilot Study

**DOI:** 10.3390/ijerph22101579

**Published:** 2025-10-16

**Authors:** Erin Speiser, Peggy-ita Obeng-Nyarkoh, Wanting Zhai, Adana A. M. Llanos, Jennifer Hicks, Chiranjeev Dash, Lucile L. Adams-Campbell, Gail E. Starr, Traci N. Bethea

**Affiliations:** 1 The Deirdre Imus Environmental Health Center^®^, Hackensack University Medical Center, Hackensack, NJ 07601, USA; 2 Geisel School of Medicine at Dartmouth, Hanover, NH 03755, USA; pao37@georgetown.edu; 3 Cancer Prevention and Control Program, Georgetown Lombardi Comprehensive Cancer Center, Washington, DC 20057, USA; wz142@georgetown.edu (W.Z.); tb988@georgetown.edu (T.N.B.); 4 Department of Epidemiology, Mailman School of Public Health, Columbia University, New York, NY 10032, USA; al4248@cumc.columbia.edu; 5 Herbert Irving Comprehensive Cancer Center, Columbia University, New York, NY 10032, USA; 6 Office of Minority Health and Health Disparities Research, Georgetown Lombardi Comprehensive Cancer Center, Washington, DC 20003, USA; 7 The Betty Torricelli Institute for Breast Care, Hackensack University Medical Center, Hackensack, NJ 07601, USA

**Keywords:** endocrine-disrupting chemicals, personal care products, environmental health, Black, Hispanic, breast cancer, cancer survivorship

## Abstract

This pilot study explored the feasibility and acceptability of utilizing silicone wristbands to assess exposure to endocrine-disrupting chemicals (EDCs) among 25 Black and Hispanic breast cancer survivors recruited in Washington, DC, and Hackensack, NJ. Over half of participants (58%) were diagnosed with Stage I breast cancer and the mean age was 58 ± 9 years. Most of the 24 survey respondents (95.83%) reported that the wristband did not interfere with daily activities and few (4) removed the wristband during the 7-day data collection period, demonstrating feasibility of use. Acceptability of passive sampling via silicone wristband was high with 73.91% of survivors reporting being “very satisfied” and 21.74% reporting being “satisfied” with their experience. The wristbands were analyzed via gas chromatography mass spectrometry for approximately 1500 semi-volatile organic compounds. This untargeted approach detected sixty distinct chemicals with an average of 21.8 per wristband. Personal care product, flame retardant, commercial product, and pesticide chemical classifications were detected in every wristband and frequently detected chemicals included biologically active compounds with potential genotoxic or endocrine-disrupting effects. This study demonstrates the feasibility of use and technical feasibility, as well as the acceptability, of utilizing silicone wristbands to assess exposure to semi-volatile organic compounds, including EDCs, among Black and Hispanic breast cancer survivors and lays the foundation towards engaging diverse cancer survivors in environmental health research.

## 1. Introduction

Silicone wristbands have been used for over a decade as personal passive samplers in observational studies assessing external exposures to an array of environmental chemicals [1,2,3,4,5]. These devices are an integral part of assessing the exposome, or the total internal and external exposures across the human lifespan which contribute to the environmental causes of disease [1]. Experimental silicone wristbands mirror conventional silicone wristbands, allowing researchers to reach a broad range of populations for study without the need for on-site infrastructure to support their use [6,7]. Silicone wristbands are known to capture dermal and inhalation exposures for semi-volatile organic compounds (SVOCs), which encompass several chemical classes present in personal care products and allow for the assessment of co-occurring exposures [8,9]. Silicone wristbands avoid the intrusive and resource-intensive nature of biospecimen-related human biomonitoring techniques, such as urine and blood collection [5]. Further, silicone wristbands can serve as an accessible method for reaching underserved populations in longitudinal research to more effectively evaluate health disparities related to environmental chemical exposure [10,11]. While chemicals in personal care products present a potential risk for environment-related disease, Black and Hispanic women use personal care products more frequently than White women and are disproportionately exposed to the chemical constituents of these products [12,13,14,15,16]. Many chemicals in personal care products have been linked to adverse health outcomes, including impacts on fertility and pregnancy, neurological issues, metabolic disease, and cancer [17,18,19,20,21,22,23]. Few studies explore PCP use and related chemical exposures among Black and Hispanic breast cancer survivors [24,25,26]. To our knowledge, this is the first study utilizing wristband sampling among breast cancer survivors in two densely populated, urban areas of northern NJ and Washington, DC.

Endocrine-disrupting chemicals (EDCs) alter the functioning of the endocrine system, which can contribute to a cascade of adverse health outcomes from obesity to cancer to hormone disruption at critical developmental timepoints [17]. Exposure to EDCs often occurs via dermal and inhalation pathways through food and personal care products [17]. Analysis of EDC exposure levels reported in the National Health and Nutrition Examination Surveys (2007–2010) found higher exposure and disease burden levels among Non-Hispanic Black and Mexican American individuals relative to the total population [27]. Discussion of exposures to EDCs and other chemicals and potentially associated health risks is not routinely part of clinical care for breast cancer survivors either during treatment or during survivorship.

The inter-method reliability of silicone wristband sampling versus biomarkers has been demonstrated in correlation studies utilizing spot urine samples [28,29,30,31]. Comparison research involving household dust samples, hand wipes, and urinary biomarkers have also found the strongest correlation between passive silicone samplers and spot urine samples, demonstrating the effective use of silicone wristbands for evaluating complex exposures of the exposome [31].

Thus, the aim of the present study was to explore the feasibility and acceptability of utilizing silicone wristbands to assess exposure to EDCs in a pilot study of Black and Hispanic breast cancer survivors.

## 2. Materials and Methods

### 2.1. Participant Recruitment

This observational cross-sectional study utilized convenience sampling to recruit Black and Hispanic breast cancer survivors who met the eligibility criteria, which were: 1. self-identify as Black/African American and/or Hispanic/Latina; 2. age ≥ 21 years; 3. history of primary breast cancer (stage I, II, or III); 4. within 5 years of completing breast cancer treatment except for endocrine therapy, if indicated; 5. able to read and speak English; 6. able to provide written consent; and 7. willing to attend two study visits, one week apart. Twenty-five breast cancer survivors aged ≥ 21 years were enrolled at a community-based site, the Office of Minority Health and Health Disparities Research (OMH) of the Georgetown Lombardi Comprehensive Cancer Center (LCCC) in Washington, DC, and from a clinic-based site, The Betty Torricelli Institute for Breast Care outpatient mammography clinic, part of Hackensack University Medical Center in Hackensack, NJ. Recruitment took place during the SARS-CoV-2 pandemic from November 2020 through December 2021.

In Washington, DC, screening for participants was conducted via review of a database of patients and volunteers who consented to being contacted about participation in research studies, as well as outreach to breast cancer survivors who previously participated in research studies and provided consent for re-contact, followed by telephone screening by study staff to confirm eligibility. Study fliers were also distributed at community outreach events and shared electronically with community partners.

In New Jersey, screening for potential participants was conducted via electronic medical records (EMR) review among women scheduled for routine mammography visits. Survivors identified via EMR review were approached by study staff in-person during their in-clinic mammography appointment. Recruitment flyers were also made available in the waiting room and mammography center staff were briefed on study recruitment to assist with identifying potential participants. Pre-screened, potential participants were approached by the Research Coordinator during regular mammogram screening appointments. Each participant was presented with the study flier and asked about interest in learning more about the study. If the participant was undecided, she was given the study flier which contained the eligibility criteria and contact information for study staff. Potential participants who expressed initial interest in the study completed an eligibility questionnaire which was administered by the Research Coordinator.

### 2.2. Data Collection

Once eligibility was confirmed and informed consent was provided, the first study visit was conducted. Participants completed the informed consent process and were provided a self-administered survey collecting data on demographics, breast cancer diagnosis, personal care product use, and potential covariates. During this session, participants were also provided with a silicone wristband for passive sampling of SVOCs and an actigraph for tracking physical activity and sleep–wake cycles [9,10]. Participants put on both items during the visit and were instructed by the research coordinator at each site to wear the silicone wristband and actigraph continually for one week and to return them at the second, final study visit. The wristbands looked and felt similar to other commercially available silicone wristbands and were designed to be lightweight and unobtrusive for wearers [6]. They were stored in sealed, specialized pouches supplied by MyExposome, Inc. prior to and after participant wear to avoid contamination outside of sampling timeframes. The three-part pouch system consisted of a small Teflon bag with a pin and clip sealing system [7] which has been extensively tested to ensure no contamination of the sample (Marc Epstein, e-mail communication, September 2025). A second study session was scheduled at the participant’s convenience for approximately seven days later. The first study visit took approximately 25–30 min.

The second study session, which lasted approximately 10–15 min, consisted of the participant returning the actigraph and the silicone wristband; completing the final survey, which included feasibility and acceptability measures; and receiving compensation. After each participant returned the actigraph, its data was downloaded using ActiLife 6 software for later analysis [32]. Each actigraph was then re-set, cleaned with an alcohol wipe, and charged for the next participant.

To assess feasibility, participants were asked, “Did you remove the silicone wristband at any time during the week?” (response choices: yes/no). Participants were asked to report how often and typical duration of any time they removed the silicone wristband. For acceptability, patients were asked, “Did the silicone wristband interfere with your daily activities?” (response choices: yes/no) and “How satisfied were you with your experience using the silicone wristband?” (response choices: very satisfied, satisfied, dissatisfied, very dissatisfied). Participants were also asked, “Were the instructions on how to use the silicone wristband clear?” and “Was the silicone wristband easy to use?” (both with response choices: yes/no).

### 2.3. Untargeted Analysis of Silicone Wristbands

As each silicone wristband was returned, it was placed back in its original, three-part pouch, which was immediately resealed and then sent via postal mail for untargeted analysis by MyExposome, Inc. [6]. Specifics of untargeted analysis methods for silicone wristbands have previously been reported [3,33]. In brief, this three-part method utilizes pre-cleaning (prior to use), infusion (exposures in the field) and laboratory-based extraction to measure an individual’s bioavailable exposures [3]. Therefore, each wristband was segmented and a section was chemically digested and analyzed using a gas chromatograph-mass spectrometer (GC-MS) for approximately 1500 chemical contaminants [34], including those commonly found in commercially available personal care products [6,35]. As the wristbands were pre-cleaned to ensure the absence of extraneous chemicals prior to shipment for study use, the untargeted GC-MS analysis method detects a range of chemicals of concern, including chemicals in personal care products, pesticides, PAHs, phthalates and volatile organic compounds [3]. Sample concentrations of detected chemicals were adjusted according to wear time and wristband size to account for accumulation of chemicals during the sampling time frame [3].

### 2.4. Statistical Data Analysis

The distribution of participant characteristics, SVOC exposure, and personal care product use was described using count and frequency for categorical variables and mean and standard deviation for continuous variables. For feasibility and acceptability, we compared survey responses by participant characteristics using Fisher exact tests due to the small sample size. One survivor was unable to complete the second study visit. Thus, analyses using data from the second study visit survey include a sample of 24 breast cancer survivors. All analyses were conducted in SAS 9.4 (SAS Institute, Cary, NC, USA).

### 2.5. Human Subject Protection

The study was conducted according to the International Conference on Harmonization (ICH), Good Clinical Practice (GCP), and the Declaration of Helsinki and in accordance with the U.S. Code of Federal Regulations on Protection of Human Rights (21 CFR 50). It was approved by the Georgetown-MedStar Institutional Review Board under Pro# STUDY00002424 and the Hackensack Meridian Health (HMH) Institutional Review Board under Pro# 2020-0999. Written informed consent was obtained from each participant prior to entering the study.

## 3. Results

### 3.1. Demographic Characteristics of Participants

Thirteen survivors were recruited in Washington, DC, and 12 survivors were recruited in Hackensack/Maywood, NJ. As shown in Table 1, the convenience sample of 25 breast cancer survivors included 17 Black and 8 Hispanic participants. Most (58%) participants had been diagnosed with Stage I breast cancer, with 7 (28%) and 4 (16%) having been diagnosed with Stage 2 or Stage 3 breast cancer, respectively. The mean age was 58 years and, on average, 4.4 years had passed since survivors’ last cancer treatment. The majority of survivors (64%) had earned a bachelor’s degree or higher, while 16% had completed high school or GED (General Educational Development). Twenty percent (5 participants) had some college with no degree, or an Associate Degree.

### 3.2. Feasibility and Acceptability of Data Collection via Silicone Wristband

Overall, self-reported compliance remained high among participants for the one-week duration of wearing the silicone wristband for passive sampling of SVOCs. Most participants (83.3%) indicated that they did not remove the silicone wristband at any time during the week of their study participation and feasibility did not differ by participant characteristics (Table 2). Similarly, 23 of the 24 survivors (95.83%) reported that the silicone wristband did not interfere with their daily activities. For acceptability most participants (73.91%) reported being “very satisfied” with their experience using the silicone wristband, while 5 (21.74%) were “satisfied” and 1 (4.35%) was “dissatisfied.” Satisfaction was poorer among participants with lower socioeconomic status (Table 3), but similar across other participant characteristics.

For the other measures of acceptability, all 24 participants (100%) reported that the instructions on how to use the silicone wristbands were clear and that the wristband was easy to use.

### 3.3. Exposure to Semi-Volatile Organic Compounds

**Figure 1:**
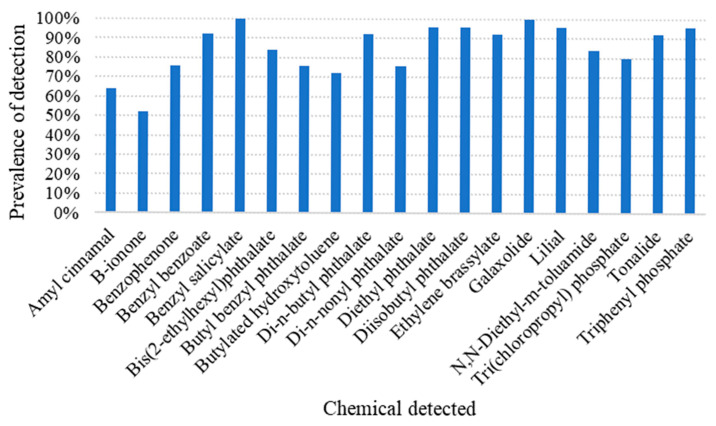
Prevalence of the chemicals detected among at least 50% of the study participants.

At study completion, the silicone wristbands from participants enrolled at both locations were shipped for analysis. Sixty distinct chemicals were detected across the 25 wristbands, with an average of 21.8 chemicals per wristband. Flame retardants and pesticides were detected in every wristband and more than half the samples were positive for the same 19 chemicals (Figure 1). Only 2 chemicals—benzyl salicylate and galaxolide—were detected in all of the wristbands (Figure 1; Table 4). Eight chemicals were detected in more than 90% of wristbands: benzyl benzoate, di-n-butyl phthalate, diethyl phthalate, diisobutyl phthalate, ethylene brassylate, lilial, tonalide, and triphenyl phosphate. The most frequently detected chemicals included biologically active compounds which are potentially genotoxic or endocrine disruptors and are often found in personal care and consumer products: benzyl salicylate [36,37], diisobutyl phthalate [38], and lilial [39]. Table 4 shows the distribution of the 19 most prevalent chemicals.

### 3.4. Self-Reported Personal Care Product Use

In the second study visit survey assessing common exposure sources for EDCs, self-reported use of perfume (52%), make-up (80%), and nail polish (68%) was prevalent during the week that participants were enrolled in the research study. Sixty percent also reported daily use of facial creams, lotions, or moisturizers, while 68% had used body creams, lotions, or moisturizers. The majority (64%) reported drinking bottled water every day or most days (24%); eating from a fast-food restaurant at least once per week (88%); and eating food reheated in a plastic container at least once per week (71%). Most participants reported never using pesticides indoors (76%) or outdoors (79%).

## 4. Discussion

Our results demonstrate the feasibility and acceptability of using silicone wristbands for passive sampling among Black and Hispanic breast cancer survivors. Exposure to environmental chemicals from personal care and other commercially available products was universal among this convenience sample of 25 Black and Hispanic breast cancer survivors living in Washington, DC and New Jersey. These findings support other research reporting that women of color are exposed to EDCs and that use of personal care products is prevalent [13]. One study evaluating the safety of personal care products which found that non-Hispanic Black women were twice as likely as non-Hispanic White women to have recently used hair products with a high hazard score on the Environmental Working Group Skin Deep^®^ database rating system [40].

While research on windows of susceptibility for exposures to chemicals in personal care products has traditionally focused on adolescence and reproductive-age/pregnancy [41,42,43,44,45,46,47,48], cancer survivors have a unique, increased susceptibility for developing secondary cancers. To our knowledge, ours is the first study on wristband sampling among breast cancer survivors in two densely populated, urban areas of northern NJ and Washington, DC. Taking into account prior wristband sampling research among other populations, it is not surprising to find ubiquitous exposures among this population.

This study reinforces the need for interventions among minority breast cancer survivors regarding practical ways to effectively reduce exposures. Such conversations do not currently fit into the typical clinical visit within essential review of tests and treatment options. Moreover, current medical education rarely covers the need for educating patients on selecting healthier personal care products and the potential downstream effects of these purchases [49]. A study such as ours serves as a springboard for finding methods to connect with oncology patients in innovative ways within the clinical framework.

As part of this study, our NJ research team met with patients as they were awaiting their routine mammograms. The outpatient clinic setting allowed time for research staff to approach potential participants and answer questions in the waiting room. The setting and timeframe represent an opportunity to teach and connect—between health educators and patients—that both parties would not otherwise have access to. A potential pool of environmental health educators may be found in training pre-med, nursing or health sciences students with the potential to create an environmental health corps at oncology service sites in diverse communities, starting with the most vulnerable. Streamlined, culturally and linguistically tailored materials could serve as the foundation for teaching the importance of choosing healthier personal care products as an essential, medically valid part of cancer survivors’ self-care.

The potential of this intervention is supported by “in the field” impressions from our research team on the openness of this group to reducing EDC exposures through healthier personal care product choices. Several participants voiced that they were eager to learn more, and had questions about what they could do to better ensure their long-term health. While this study did not include a formal teaching component, the questions and discussions that participants had after data collection was complete, reinforced that educational intervention is a much-needed next step.

Overall, survivors stand to benefit from increased research and interventions to lower potential EDC exposures as a cancer prevention mechanism. The need for educational interventions on EDC exposures among this population potentially provides increased opportunities for engaging breast cancer survivors through the healthcare framework to provide ongoing education on reducing exposure to EDCs, as well as biomonitoring opportunities to measure the effectiveness of interventions.

In this study, passive sampling of environmental exposures using a silicone wristband was well tolerated for the one-week study duration, suggesting this method could be expanded as part of an exposome-wide approach to tertiary cancer prevention [50]. The simplicity of instructions and ease of use for the silicone wristband suggest that this method of passive sampling could be utilized in future research among a larger population of Black and Hispanic breast cancer survivors. Unlike other human biomonitoring methods with potentially higher research burden, utilization of wristbands requires that study staff receive a brief training prior to deploying this sampling method and the small size of the wristbands makes compact storage and deployment in a busy clinical or research setting ideal.

Future research is warranted to investigate the role of endocrine-disrupting chemicals, particularly those from personal care products, as well as educational interventions within the clinical oncology framework, among Black and Hispanic breast care survivors to reduce cancer health disparities.

### Strengths and Limitations

The limitations of utilizing silicone wristbands include inability to differentiate between dermal and inhalation exposure pathways [2]. Wristbands also represent a time-weighted average, not an episodic concentration and cannot measure chemicals absorbed through oral exposures [51]. Mechanisms of chemical accumulation into the silicone wristbands remain largely unexplored, though recent research is contributing to a growing understanding of how factors such as the wearers’ movements affect sampling outcomes [52].

Despite the widespread disruptions of the COVID-19 pandemic during recruitment for this study, the two research teams were able to enroll participants while following pandemic guidelines in place at both healthcare organizations. While the initial plan included four months for patient recruitment, the pandemic and the need to add a second study site stretched the enrollment period to eleven months. Due to the small sample size, this research has limited generalizability to other Black and Hispanic breast cancer survivors living outside the catchment area of the Georgetown Lombardi Comprehensive Cancer Center in Washington, DC and The Betty Torricelli Institute for Breast Care in New Jersey.

Subject recruitment was conducted via convenience sampling during the COVID-19 pandemic, which may have introduced bias associated with patients who chose to attend the in-person appointments and survivors who were not essential workers. This may limit the generalizability of the findings as some cancer survivors may have opted to delay appointments due to pandemic-related concerns and the convenience sample reported high socioeconomic status. Additionally, social desirability bias may have impacted participant responses, although rapport with the study team, which was established during recruitment and assessment of eligibility, may have minimized its impact. Considering these limitations, our study still provides preliminary evidence that breast cancer survivors are willing and able to participate in exposure assessment via passive sampling, which may inform larger future studies to investigate and reduce exposures to environmental chemicals in this population.

## 5. Conclusions

This study demonstrates the feasibility and acceptability of utilizing silicone wristbands to assess exposure to semi-volatile organic compounds, including endocrine-disrupting chemicals, among Black and Hispanic breast cancer survivors. Overall, study participants were highly engaged and willing to share their experiences. This study lays the foundation towards engaging cancer survivors, who are often excluded from eligibility in environmental health studies, in future research and leveraging educational interventions to reduce EDC exposure through choosing healthier personal care products to help empower cancer survivors. These may be developed within the clinical framework, via training pre-med, nursing and health sciences students to deliver vital environmental health information and engage oncology patients to take an active role in health outcomes.

## Figures and Tables

**Table 1:** Sociodemographic factors among the 25 pilot study participants at baseline.

Characteristics	*N* [%] or Mean ± Std Dev
Age, years	57.76 ± 9.12
<50	5 [20]
50–60	12 [48]
≥60	8 [32]
Race/ethnicity	
Black/African American, non-Hispanic	17 [68]
Hispanic/Latinx	8 [32]
Marital status	
Single, never married	10 [40]
Married/live with a partner/live as married	11 [44]
Divorced/separated/widowed	4 [16]
Highest education attained	
High school diploma/GED	4 [16]
Some college, no degree/Associate Degree	5 [20]
Bachelor’s degree or higher	16 [64]
Total family income, U.S. Dollars	
<$10,000	2 [8]
$10,000–$34,999	2 [8]
$35,000–$49,999	4 [16]
$50,000–$74,999	2 [8]
≥$75,000	13 [52]
Missing	2 [8]
Currently employed	
Yes	16 [64]
No	9 [36]

**Table 2:** Feasibility of passive data collection via silicone wristband among 24 breast cancer survivors by participant characteristics.

Characteristics	Did You Remove the Silicone Wristband at Any Time During the Week?		
	No (*n* = 20) *n* [%]	Yes (*n* = 4) *n* [%]	*p*
Age (years)			0.64
<50	5 [20.8]	0	
50–60	10 [41.7]	2 [8.3]	
≥60	5 [20.8]	2 [8.3]	
Race/ethnicity			>0.90
Black/African American	14 [58.3]	3 [12.5]	
Hispanic/Latinx	6 [25.0]	1 [4.2]	
Marital status			0.10
Single, never married	9 [37.5]	0	
Married/live with a partner/live as married	9 [37.5]	2 [8.3]	
Divorced/separated/widowed	2 [8.3]	2 [8.3]	
Highest education attained			0.74
High school diploma/GED	3 [12.5]	0	
Some college, no degree/Associate Degree	5 [20.8]	0	
Bachelor’s degree or higher	12 [50.0]	4 [16.7]	
Total family income, U.S. Dollars			0.45
<$35,000	3 [12.5]	0	
$35,000–$49,999	4 [16.7]	0	
≥$50,000	12 [50.0]	3 [12.5]	
Missing	1 [4.2]	1 [4.2]	
Currently employed			0.58
Yes	14 [58.3]	2 [8.3]	
No	6 [25.0]	2 [8.3]	

All *p*-values are from Fisher’s exact tests. Frequencies may not total 100% due to rounding.

**Table 3:** Acceptability of passive data collection via silicone wristband among 23 breast cancer survivors by participant characteristics.

Characteristics	How Satisfied Were You with Your Experience Using the Silicone Wristband?			
	Very Satisfied (*n* = 17) *n* [%]	Satisfied (*n* = 5) *n* [%]	Dissatisfied (*n* = 1) *n* [%]	*p*
Age (years)				0.63
<50	3 [13.0]	1 [4.4]	1 [4.4]	
50–60	9 [39.1]	2 [8.7]	0	
≥60	5 [21.7]	2 [8.7]	0	
Race/ethnicity				0.37
Black/African American	13 [56.6]	3 [13.0]	0	
Hispanic/Latinx	4 [17.4]	2 [8.7]	1 [4.4]	
Marital status				0.48
Single, never married	6 [26.1]	1 [4.4]	1 [4.4]	
Married/live with a partner/live as married	7 [30.4]	4 [17.4]	0	
Divorced/separated/widowed	4 [17.4]	0	0	
Highest education attained				0.01
High school diploma/GED	1 [4.4]	1 [4.4]	1 [4.4]	
Some college, no degree/Associate Degree	2 [8.7]	3 [13.0]	0	
Bachelor’s degree or higher	14 [60.9]	1 [4.4]	0	
Total family income, U.S. Dollars				0.13
<$35,000	1 [4.4]	1 [4.4]	0	
$35,000–$49,999	2 [8.7]	1 [4.4]	1 [4.4]	
≥$50,000	13 [56.5]	2 [8.7]	0	
Missing	1 [4.4]	1 [4.4]	0	
Currently employed				0.05
Yes	14 [60.9]	2 [8.7]	0	
No	3 [13.0]	3 [13.0]	1 [4.4]	

All *p*-values are from Fisher’s exact tests. Counts add to 23 due to 1 participant not providing data on satisfaction. Frequencies may not total 100% due to rounding.

**Table 4:** Distribution of chemicals detected among at least 50% of the study participants (in nanograms per gram of wristband).

Variable	Mean	Std Dev	Minimum	Maximum	Median
Amyl cinnamal	115.83	128.97	0.00	437.00	86.10
B-ionone	115.03	149.67	0.00	525.00	69.30
Benzophenone	169.74	148.04	0.00	549.00	158.00
Benzyl benzoate	6294.96	7652.96	0.00	28,100.00	2750.00
Benzyl salicylate	6695.32	6551.87	331.00	25,500.00	4140.00
Bis(2-ethylhexyl)phthalate	23,063.84	30,730.56	0.00	121,000.00	12,900.00
Butyl benzyl phthalate	829.48	1426.69	0.00	6720.00	384.00
Butylated hydroxytoluene	86.87	141.76	0.00	522.00	33.20
Di-n-butyl phthalate	2549.84	1586.93	0.00	6450.00	2320.00
Di-n-nonyl phthalate	574.60	1049.19	0.00	5250.00	312.00
Diethyl phthalate	2773.40	5230.53	0.00	25,800.00	1100.00
Diisobutyl phthalate	3235.80	4494.12	0.00	21,500.00	1800.00
Ethylene brassylate	4923.08	6534.55	0.00	29,700.00	2810.00
Galaxolide	6928.00	6562.93	139.00	20,100.00	3850.00
Lilial	446.34	581.03	0.00	2790.00	273.00
N,N-Diethyl-m-toluamide	1615.93	5883.23	0.00	29,700.00	215.00
Tri(chloropropyl)phosphate	1096.23	3406.83	0.00	16,200.00	209.00
Tonalide	446.67	721.12	0.00	3330.00	188.00
Triphenyl phosphate	450.83	485.70	0.00	2030.00	233.00

Values were normalized to wear time. Values that were unable to be quantified were treated as zeroes.

## Data Availability

The datasets used for this study are available from the corresponding author on reasonable request subject to IRB approval.

## Abbreviations

The following abbreviations are used in this manuscript:

EDCs	Endocrine-disrupting chemicals
PCP	Personal care product
SVOCs	Semi-volatile organic compounds
